# Passive Immunization Delays Disease Outcome in Gilthead Sea Bream Infected With *Enteromyxum leei* (Myxozoa), Despite the Moderate Changes in IgM and IgT Repertoire

**DOI:** 10.3389/fimmu.2020.581361

**Published:** 2020-09-11

**Authors:** Amparo Picard-Sánchez, Itziar Estensoro, Pedro Perdiguero, Raquel del Pozo, Carolina Tafalla, M. Carla Piazzon, Ariadna Sitjà-Bobadilla

**Affiliations:** ^1^Fish Pathology Group, Institute of Aquaculture Torre de la Sal (IATS-CSIC), Castellón, Spain; ^2^Centro de Investigación en Sanidad Animal (INIA), Madrid, Spain

**Keywords:** *Sparus aurata*, passive immunity, antibodies, intestinal parasite, immune response, immunoglobulin repertoire

## Abstract

Passive immunization constitutes an emerging field of interest in aquaculture, particularly with the restrictions for antibiotic use. *Enteromyxum leei* is a myxozoan intestinal parasite that invades the paracellular space of the intestinal epithelium, producing a slow-progressing disease, leading to anorexia, cachexia and mortalities. We have previously demonstrated that gilthead sea bream (GSB, *Sparus aurata*) that survive *E*. *leei* infection become resistant upon re-exposure, and this resistance is directly related to the presence of high levels of specific IgM in serum. Thus, the current work was aimed to determine if passive immunization could help to prevent enteromyxosis in GSB and to study in detail the nature of these protective antibodies. Serum from a pool of resistant (SUR) or naïve (NAI) animals was intracoelomically injected 24 h prior to the *E*. *leei*-effluent challenge and at 9 days post-challenge (dpc). Effluent challenge lasted for 23 days, and then the injected groups were allocated in separate tanks with clean water. A non-lethal parasite diagnosis was performed at 56 dpc. At the final sampling (100 dpc), blood, serum and tissues were collected for histology, molecular diagnosis and the detection of circulating antibodies. In parallel, we performed an immunoglobulin repertoire analysis of the fish generating SUR and NAI sera. The results showed that, fish injected with parasite-specific antibodies (spAbs) became infected with the parasite, but showed lower disease signs and intensity of infection than the other groups, indicating a later establishment of the parasite. Repertoire analysis revealed that *E. leei* induced a polyclonal expansion of diverse IgM and IgT subsets that could be in part an evasion strategy of the parasite. Nonetheless, GSB was able to produce sufficient levels of parasite-spAbs to avoid re-infection of surviving animals and confer certain degree of protection upon passive transfer of antibodies. These results highlight the crucial role of spAb responses against *E. leei* and set the basis for the development of effective treatment or prophylactic methods for aquaculture.

## Introduction

Pathogens are an important cause of economic losses in aquaculture, and the partial effectiveness of the available treatments increases the chances of drug-resistant pathogens in fish and in the aquatic environment (1). The therapeutic use of specific antibodies (spAbs) is an attractive alternative to provide immunity against pathogens. Opposite to chemicals and antibiotics, which have a broad-spectrum of action, antibodies constitute a very specific defense mechanism. Antibody mediated immunity can be active or passive. Active immunity depends on the production of spAbs after direct contact with the pathogen or antigen (vaccination or random encounter), it takes days/weeks to develop and results in the formation of immunological memory that can last for months or years. In passive immunity, spAbs obtained from a previously infected or immunized donor, are introduced in a naïve individual to confer resistance or to combat a specific pathogen. Passive immunity is faster, short-lived and does not involve memory (2).

Passive immunization can be allogeneic or xenogeneic, if the origin of the transferred Abs is the same or different than the recipient species, respectively. This technique has been applied successfully in human medicine (3). It has also been tested in fish-parasite models showing promising results. Allogeneic therapies have partially protected carp (*Cyprinus carpio*) against *Trypanoplasma borreli* (4), or tomato clownfish (*Amphiprion frenatus*) against *Amyloodinium ocellatum* (5). However, passive immunization failed to protect in other fish-parasites models like rainbow trout (*Oncorhynchus mykiss*) against *Gyrodactylus derjavini* infection (6). Of note, the first two experiments required repeated doses of serum to induce protection, whereas in the latter, only one dose was administered. Xenogeneic therapies have also been applied successfully. Mouse monoclonal antibodies against surface proteins of *Ichthyophthirius multifiliis* protected channel catfish (*Ictalurus punctatus*) against infection (7). Clearly, a key aspect for the success of the procedure is a deep knowledge about the host-parasite model, especially the time for parasite spreading in the host and the time that transferred antibodies remain in the circulation of the recipient animal (8).

Teleost fish have three different immunoglobulin (Ig) isotypes: IgM, IgT/Z, and IgD (9). The basic structure of fish Igs is the same as that of mammals. The heavy chain is constituted of an isotype-specific constant domain (CH) and a variable domain (VH) which, together with the variable domain of the light chain (VL), are responsible for the great diversity of antigen binding sites. Each V domain has three hyper variable regions termed complementarity-determining regions (CDR), which constitute the majority of the antigen binding sites of the Ig molecule. Different combinations of CDR in VL and VH are responsible for the amazing diversity in binding sites among the millions of antibodies in an individual (10). In fish, IgM is the highest expressed Ig in all organs and it is essential for immune protection against different pathogens upon different routes of infection (11–15). IgM is highly abundant in fish serum with concentrations between 800 and 9,000 μg/ml (16). The teleost specific isotype IgT is considered the most important Ig in mucosal surfaces, but is also found in serum at much lower concentrations than IgM, so its role in systemic responses should not be discarded (17–20). The role of IgD is still not well defined in mammals or fish, however, recent studies performed in fish have established that it is also secreted (21) and might have a relevant role in some mucosal surfaces such as gills (18) or intestine (22). Like mammals, fish can develop immunological memory. A secondary exposure to an antigen will induce faster and greater responses than primary response, with larger numbers of antigen-specific B cells originated from the expansion of the pool of memory B cells. Detailed repertoire analyses have helped to define fish Ig responses upon infections of different etiology (17, 23).

*Enteromyxum leei* is a myxozoan intestinal endoparasite, that can be transmitted from fish-to-fish and causes a slow progressing catarrhal enteritis in gilthead sea bream (GSB, *Sparus aurata*) (24, 25). The parasite lives and divides in the paracellular space between enterocytes causing chronic infections with intestinal inflammation leading to dysfunctional absorption and anorexia, reflected in weight loss and decreased specific growth rate (SGR), condition factor (CF), and fat deposits in liver (25). Currently, this parasite cannot be cultured *in vitro* and there are no preventive or curative measures against this disease. However, there is plenty of background knowledge on the host response against the parasite (25–31). We have recently demonstrated that GSB is able to develop acquired immunity against *E*. *leei*. Fish that survived an infection did not get re-infected upon re-exposure, even 16 months after the initial exposure, and this resistance was correlated with high levels of spAbs (IgM) in serum (29). Therefore, the aim of the present study was to demonstrate the protective role of spAbs against enteromyxosis in GSB by passive immunization of naïve animals. We also aimed to characterize the type of Ig response produced by this parasite, analyzing and comparing the Ig repertoire in naïve and resistant animals re-exposed to the parasite. The results obtained in this study provide novel information to understand the role of antibodies during the response of GSB to enteromyxosis, and will lead us a step further toward developing successful vaccines and/or treatments against this economically important disease in aquaculture.

## Materials and Methods

### Fish Stock and Maintenance

Specific-pathogen-free (SPF) and clinically healthy GSB juveniles from a commercial fish farm, were kept in 5 μm-filtered and UV irradiated sea water (salinity 37.5 g/l) between 18 and 21.8°C, with the natural photoperiod at latitude 40à5′N; 0à10′E. The SPF status was confirmed by qPCR according to the protocol previously described (29). Fish were fed *ad libitum* once a day a commercial diet (BioMar, Palencia, Spain). All experimental protocols involving fish were approved by the Ethics and Animal Welfare Committee of IATS, CSIC, and Generalitat Valenciana. They were carried out in a registered installation facility in accordance with the principles published in the European animal directive (2010/63/EU) and Spanish laws (Royal Decree RD53/2013) for the protection of animals used in scientific experiments. All efforts were made to minimize the suffering of animals.

### Fish Serum and Tissues Samples

Fish sera used for the passive immunization trial were obtained from a previous experimental infection (29). Briefly, GSB that had survived and cleared an infection with *E*. *leei* were re-exposed to the parasite 9 months after the beginning of the first infection by exposure to parasite-infected water effluent. These fish showed to be resistant to re-infection (SUR) and tested negative for the presence of the parasite 61 and 86 days after the second exposure. Sera from the four fish that showed the highest levels of parasite-specific IgM (immunoreactivity = 6, see below) were selected and pooled. SUR serum and tissue samples were obtained 86 days after the second exposure to the parasite. In parallel, sera from four naïve (NAI) fish from the same batch were tested to be negative for parasite-specific IgM and pooled to be used as a control. Before injection, the complement was heat-inactivated (42àC 30 min). From the same eight animals, RNA from the anterior intestine was extracted as previously described (29). The anterior intestine was chosen because it is the tissue where immunoglobulin gene expression was significantly increased upon re-exposure to the parasite in SUR animals (29).

### Passive Immunization Trial

Fifty naïve GSB (25.5 g) were individually tagged with passive integrated transponders (PIT-tags) and kept together in the same tank during the first part of the trial involving serum injections and effluent challenge. One day before and nine days after the challenge, 20 fish per group were injected intracoelomically (i.c.) with 10 μl/g of NAI or SUR serum. The remaining 10 fish were injected with 10 μl/g of sterile PBS. In addition, 10 naïve fish from the same stock were kept separately, constituting the non-challenged control group. A preliminary trial was conducted to determine the duration of injected antibodies in the recipient fish blood circulation. For that purpose: 10 μl/g of rabbit serum (DAKO) were i.c. injected and rabbit Igs were detected by dot blot in serum as soon as 1 h post-injection and remained in circulation at least 9 days (Supplementary Figure S1).

**FIGURE 1:**
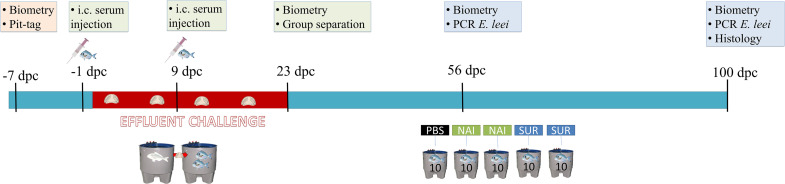
Schematic representation of the passive immunization trial. Seven days before the challenge by effluent exposure, fish were tagged, weighted, measured and allocated together in the same tank. One day before the challenge and nine days post-challenge (dpc) fish were injected with the different sera or PBS. At 23 dpc, the effluent challenge was terminated and fish were separated in three different tanks (10 fish/tank, SUR, NAI, and PBS injected groups). At 56 dpc, a non-lethal diagnosis of the parasite by PCR was performed, and at 100 dpc all fish were sacrificed and samples for histology and diagnosis were collected. i.c., intracoelomic.

One day (24 h) after the first injection, fish were challenged with *E*. *leei* by exposure to infected water effluent, mimicking one of the natural routes of infection in farmed fish (32). Briefly, the tank holding 50 fish, already injected with PBS, NAI or SUR serum, received water effluent from a donor tank holding 33 heavily *E*. *leei*-infected GSB, as previously described (33). At 23 days post-challenge (dpc), fish were weighed and measured, and the different injected groups were moved in groups of 10 to individual tanks receiving clean water, ending the effluent challenge. Serum-injected fish (*n* = 20 for each type of serum) were moved to two replicate tanks (*n* = 10) for each group. The 23-day exposure was selected as the minimum time needed for all fish to be challenged with the parasite without exerting a too high infection pressure that will mask the results at the end of the trial (34). At 56 dpc, fish were non-lethally sampled to evaluate the progression of the infection and the final lethal sampling was performed at 100 dpc. A schematic representation of the trial and samplings can be found in Figure 1.

Before all samplings (7 days before challenge, 23, 56, and 100 dpc), fish were starved for two days and biometric parameters measured. Additionally, at the second sampling (56 dpc), fish were also sampled for parasite diagnosis by non-lethal rectal probing and qPCR with specific primers for the parasite 18S rRNA gene, as previously described (29). Finally, in the last sampling (100 dpc), fish were killed by overexposure to anesthetic (MS-222, 0.1 g/l; Sigma), and blood and tissue samples for molecular and histological procedures were taken. Blood samples (1 ml) were drawn from the caudal vessels by puncture with heparinized sterile needles and serum was obtained after overnight clotting at 4°C and centrifugation at 3,000 × *g* for 30 min, and maintained at -80°C until further use. Intestine (anterior-immediately after the pyloric caeca-, middle and posterior-immediately before the anal ampoule), head kidney, spleen, and liver samples from all fish were taken in 10% buffered formalin for standard histological procedures. Condition factor (CF = [100 × body weight]/fork length^3^) and specific growth rate (SGR = [ln (final weight) – ln (initial weight) × 100]/days) were calculated for each animal individually. Infection intensity was semiquantitatively evaluated on Giemsa stained histological sections of anterior (AI), middle (MI), and posterior intestine (PI) using a scale from 1 (lowest) to 6 (highest) (28). The remaining intestinal tissue was processed for molecular diagnostic (qPCR), as previously described (29). In addition, PAS-staining was carried out on liver sections. One fish of the NAI group that died between the scheduled sampling points was checked post-mortem for the presence of the parasite and was no longer included in the results.

### Circulating Antibody Detection

Total IgM and IgT were measured by ELISA as previously described (35). Specific IgM against *E*. *leei* in serum samples was immunohistochemically detected using paraffin embedded sections of *E*. *leei*-infected GSB intestines obtained from previous and independent infection trials, following an immunohistochemical sandwich ELISA protocol, as described previously (29). Intensity of immunoreactivity of each fish serum against the parasite was evaluated by microscopic examination of the immunolabelled tissue sections according to a semiquantitative scale ranging from 0 to 6 (scaling: 0 = no immunoreactivity against the parasite; 1 = very slight reactivity; 2 = slight reactivity; 3 = medium reactivity; 4 = medium-intense reactivity; 5 = intense reactivity; 6 = very intense reactivity).

### Immunohistochemistry

The detection of T cells (Zap70^+^) in the intestine of experimental individuals was performed by immunohistochemistry (IHC), as previously described (28). In the former study, we have reported that intestinal T cells increase in numbers upon *E. leei* infection and these cells are one of the key mechanisms to clear the parasite. Briefly, paraffin sections (4 μm thick) were deparaffinized, hydrated and the endogenous peroxidase activity was blocked by incubation in hydrogen peroxide (0.3% (v/v) for 30 min). An antigen retrieval step was performed by boiling the samples in citrate buffer pH 6 for 20 min. Incubations were performed in a humid chamber at room temperature and all washing procedures consisted of successive 5 min immersions in TTBS (20 mM Tris-HCl, 0.5 M NaCl, 0.05% Tween 20, pH 7.2) and TBS (20 mM Tris-HCl, 0.5 M NaCl, pH 7.2). Slides were washed, blocked for 30 min with 1.5% normal goat serum (VECTOR Laboratories). After washing, samples were then incubated with a monoclonal rabbit anti-Zap70 antibody (99F2 Cell Signaling Technologies) diluted 1:50 in TBS 1% BSA for 1 h and washed again. Slides were incubated with a biotinylated goat anti-rabbit antibody (VECTOR Labs.) diluted 1:200 in TBS 1.5% normal goat serum for 1 h, washed and the avidin-biotin-peroxidase complex (ABC) (VECTOR Labs.) was applied for 1 h before washing slides again. Bound peroxidase was developed by adding 3,3’-diaminobenzidine tetrahydrochloride chromogen (Sigma) for 2 min and the reaction was stopped with deionized water. Tissue sections were counterstained with Gill’s hematoxylin, dehydrated and mounted with di-N-butyl phthalate in xylene. Incubation of tissue sections with ABC alone served as control to discard the presence of endogenous biotin-binding proteins. Negative controls omitting the primary antibodies, the secondary antibody and the ABC were carried out and were negative.

### Immunoglobulin Repertoire Amplification and Sequencing

To perform repertoire analysis of GSB immunoglobulins, genomic regions potentially encoding IGHV genes were identified along GSB draft genome (36), searching for conserved domains using the online tool https://www.ncbi.nlm.nih.gov/Structure/cdd/wrpsb.cgi from NCBI. Transcriptional activity of identified regions were further confirmed by blasting against a GSB transcriptome from a previous study (37). Nucleotide sequences were aligned using ClustalW^1^ in order to identify conserved regions among genes to design primers which may amplify all potential genes. A total of seven forward primers, recognizing the FR3 region, were designed and used in PCR amplifications in combination with reverse primers designed in IgM or IgT constant region (Cμ2 and Cτ1, accession numbers KX599199 and KX599201, respectively) (Supplementary Table S1).

To study the IgM and IgT repertoire in the fish used as serum donors for passive immunization, cDNA from anterior intestine of SUR and NAI donor fish were used as template in PCR amplifications for each primer combination (F + R). Mix of reagents for PCR reaction was: 1 μl of cDNA as a template, 0.2 μM of each forward and reverse primer combination, and 2.5 U/μl DNA polymerase (Accuprime^TM^ Taq polymerase High Fidelity, INVITROGEN) in 1 × AccuPrime PCR Buffer I, containing 2 mM MgCl_2_ and a mix of 0.2 mM of each dNTP (INVITROGEN). The thermal cycling regime was as follows: 94°C for 2 min, followed by 40 cycles (94°C for 45 s, 55°C for 1 min, and 72°C for 1 min) and the final extension at 72°C for 10 min. Negative controls without cDNA were also included.

For the visualization of PCR products, a 10 μl aliquot from each PCR product was mixed with 2 μl of loading buffer and loaded into a 1% agarose with ethidium bromide staining. At the same time, samples for sequencing were composed by 2 μl from each reaction belonging to the same individual pooled together. Samples were quality checked using an Agilent 2100 Bioanalyzer (Agilent Technologies) and sequenced by Life Sequencing (Valencia, Spain). One library per individual was constructed with the TruSeq DNA PCR-Free Library Prep Kit (Illumina, San Diego, CA, United States) according to the manufacturer’s protocol. Libraries were pooled together, and paired-end sequencing was performed on an Illumina MiSeq with a MiSeq Reagent Kit v3 (2 × 300 cycles) cartridge (Illumina, San Diego, CA, United States).

### Sequence Analysis

Raw sequencing data were demultiplexed and sequencing adapters and barcodes were removed from the sequences using the MiSeq Analysis pipeline. The first 20 nucleotides from the reverse primers were used as a barcode for the identification of 3’ ends corresponding to each constant Ig gene. Reads that matched a primer sequence with a maximum of three mismatches allowing an overlap of two nucleotides (–mismatches 3 –partial 2) were classified into the corresponding IgM or IgT isotype using the FASTQ/A Barcode splitter tool^2^. The opposite paired end reads, corresponding to the 5′ end of PCR products, were extracted with the FASTQ interlacer tool implemented in Galaxy (38). The paired forward and reverse reads were merged using PEAR software (39) with a minimum overlap size of 10 nucleotides. Finally, a quality filter was applied keeping sequences with a phred base quality ≥20 in at least the 90% of sequence.

In the current study, in absence of a V-D-J germline for our species, sequences for each isotype from the eight individuals were annotated by comparative genomics according the germline of zebrafish (*Danio rerio*), used as model species, which is available at International Immunogenetics information system databases (40). Thus, GSB repertoire was annotated according the best match identified for each gene (V,D, or J) from zebrafish using the IMGT/HighV-QUEST tool (41). IMGT/HighV-QUEST results were parsed using convert tool from VDJtools software (42). Non-functional clonotypes were filtered out using FilterNonFunctional tool from VDJtools software. Retained functional clonotypes were further analyzed using the repExplore, repClonality, repDiversity, geneUsage, and getKmers tools from the R package immunarch (v0.5.5) (43). Prior to analysis, data were normalized by subsampling. This analysis allowed a first preliminary glimpse on the diversity of the clonotypes and the type of response that is being elicited by the parasite by comparison of two sampling groups (NAI vs. SUR).

### Statistical Analysis

Differences among groups were evaluated with one-way ANOVA followed by Tukey test. Data which failed the normality or equal variance test were analyzed with Kruskal–Wallis ANOVA-I on ranks followed by Dunn’s method. Differences between two groups were determined by Student’s *t*-test or Mann–Whitney test when normality conditions were not met. A chi-square test was run to determine differences in the presence/absence of liver fat and glycogen deposits. In all cases differences were considered significant at *P* < 0.05. Statistics and visualizations were performed with the R package immunarch (v0.5.5) (43) and GraphPad Prism (v6.01).

## Results

### Effects of Passive Immunization on Clinical Signs and Infection Parameters

**FIGURE 2:**
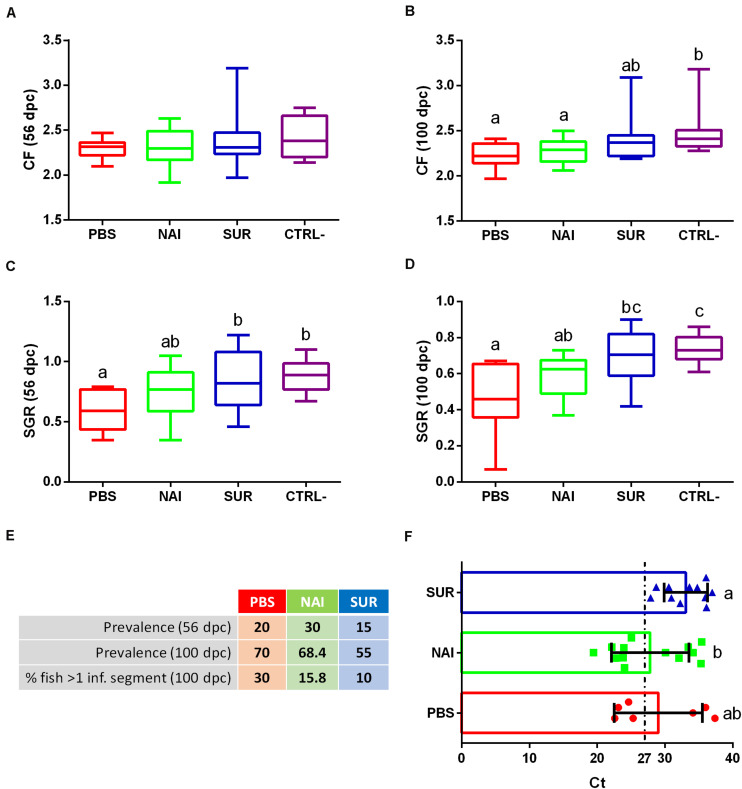
Disease signs and parasite diagnosis. Condition factor (CF), **(A,B)** and specific growth rate (SGR), **(C,D)** were calculated for the intermediate **(A,C)** and final samplings **(B,D)**. Prevalence of infection is represented as the percentage of infected fish at the intermediate and final samplings (56 and 100 days post-challenge, dpc). The percentage of fish with more than one intestinal segment parasitized (% fish > 1 inf. segment) was calculated at the final sampling **(E)**. Intensity of infection is shown as Ct values (mean ± SD) obtained from a parasite specific qPCR of the intestine at 100 dpc **(F)**. Low Ct values indicate high infection intensity. PBS is the group injected with PBS, whereas NAI represent the fish injected with serum without specific antibodies and SUR the fish injected with parasite-specific antibodies. The negative control (CTRL-) was the group not challenged with the parasite. Different letters indicate significant differences among groups (*P* < 0.05).

The typical clinical signs of enteromyxosis include weight loss and decreased CF and SGR (25). The current results show that while CF of some challenged fish was not affected until the final sampling at 100 dpc, SGR was already affected at the intermediate sampling (56 dpc) in the PBS-injected group (Figures 2A–D). Fish injected with PBS or NAI serum always showed lower CF and SGR than non-challenged fish. Interestingly, fish injected with SUR serum did not show significant differences with the non-challenged controls and always displayed intermediate values.

No significant differences were found in the prevalence of infection among the different challenged groups in any of the sampling points. However, the prevalence of infection in fish injected with parasite-spAbs (SUR serum) was always the lowest (Figure 2E). Histological diagnosis allowed assessing the percentage of fish with a progressed infection, that is, fish that have more than one intestinal segment parasitized. Progression of infection also showed lower values in fish injected with SUR serum (Figure 2E). Interestingly, all infected passively immunized fish (SUR serum) showed low intensity of infection (Ct values > 27), whereas, roughly half of the individuals from the other two groups had high intensity of infection (Ct values < 27). The intensity of infection of SUR-injected fish was significantly lower than that of the NAI serum group (Figure 2F, higher Ct values correspond to lower intensity of infection).

### Effects of Passive Immunization on Histopathology

**FIGURE 3:**
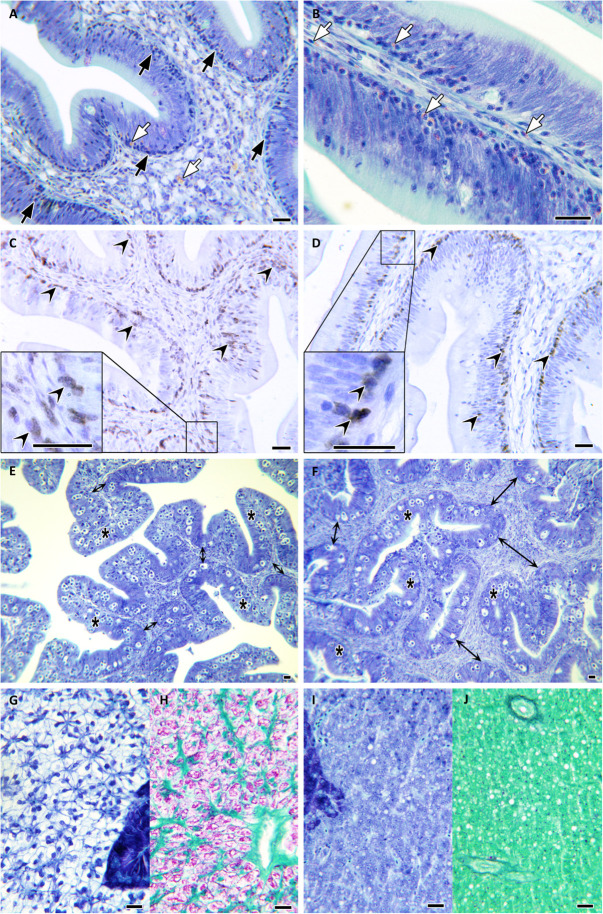
Histopathology. Microphotographs of gilthead sea bream tissue sections stained with Giemsa **(A,B,E,F,G,I)**, PAS **(H,J)**, or immunolabelled with anti-Zap70 **(C,D)**. Anterior intestinal sections of SUR-sera injected fish at 100 days post challenge **(A,B)**. Note the abundance of lymphocyte-like cells at the epithelial base and infiltrating the enterocyte layer (black arrows) and the abundant eosinophilic granular cells (white arrows). Zap70^+^ T cells at the anterior intestine of SUR-injected **(C)** and NAI-injected fish **(D)** (black arrowheads). Posterior intestine of SUR-injected **(E)** and NAI-injected fish **(F)** showing increased hyperplasia of the submucosa at the latter (double-headed arrows). Infected epithelia with *Enteromyxum leei* stages are indicated by asterisks. Comparative liver sections of SUR-injected **(G,H)** and NAI-injected fish **(I,J)**. Note the abnormal absence of fat (colorless) and glycogen (purple) deposits in the hepatocytes shown in panels **(I,J)**. Scale bars = 20 μm.

All parasite-challenged fish showed the typical intestinal reaction against *E*. *leei*, although some differences could be observed among groups. Overall, 70, 73.7, and 85% of the PBS, NAI, or SUR fish, respectively, had abundant lymphocyte-like cells at the base of the intestinal epithelium, particularly at the anterior segment (Figure 3A). IHC revealed that most of these cells were Zap70^+^, identifying, at least part of this increased cell population, as T cells (Figures 3C,D). All challenged groups showed a noteworthy infiltration of eosinophilic granular cells in the epithelial layer (Figure 3B). Interestingly, the prototypical hyperplasia of the submucosa upon *E*. *leei* infection was observed in fewer animals injected with parasite-spAbs (35% in SUR vs. 59% in NAI) (Figures 3E,F). The most striking difference was found in the liver. The liver of heavily infected fish, due to the anorexia and the nutrient absorption impairment induced by the parasite, had clearly less accumulation of fat and glycogen deposits. This was the case for most PBS (80%) and NAI (42%) challenged fish, whereas all SUR challenged fish, except one (5%), had normal liver histological features with abundant fat and glycogen deposits (Figures 3G,H,I,J). This difference was statistically significant (chi-squared test, *P* = 0.02).

### Effects of Passive Immunization on Circulating Antibodies

**FIGURE 4:**
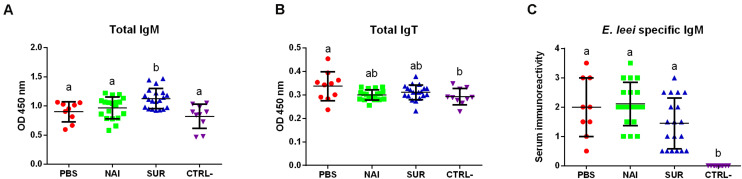
Circulating antibodies. Total IgM **(A)**, IgT **(B)**, and parasite-specific IgM **(C)** in fish injected with PBS, or with serum without (NAI) or with (SUR) parasite-specific antibodies. The negative control (CTRL-) were fish not challenged with the parasite. Different letters indicate significant differences among groups (*P* < 0.05).

Total serum IgM, IgT and parasite specific IgM of the different groups were measured at 100 dpc. Total circulating IgM was significantly higher in the group injected with serum with parasite-spAbs than that of all the other groups, which did not show differences among them (Figure 4A). Circulating IgT was higher in the PBS injected group than in the control non-challenged group. The serum injected groups showed intermediate values (Figure 4B). No parasite-specific IgM was found in the non-challenged group, while all the other groups showed no differences among each other (Figure 4C).

### Immunoglobulin Repertoire Analysis

**FIGURE 5:**
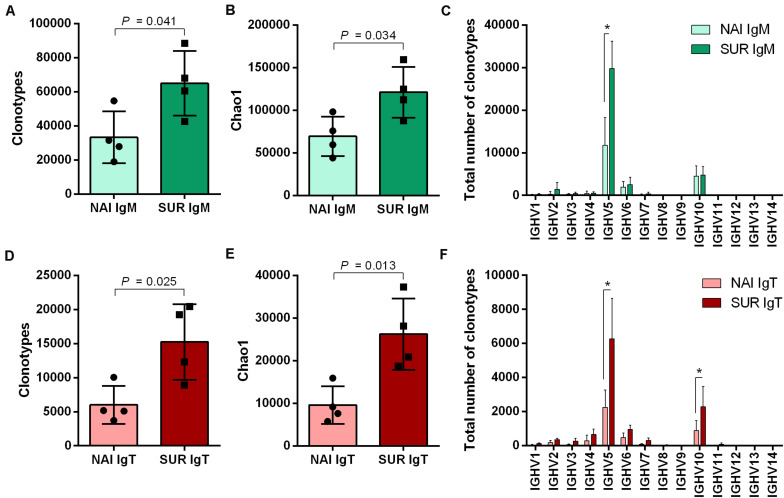
Clonotype diversity and V family usage. Number of unique IgM **(A)** and IgT **(D)** clonotypes detected in the anterior intestine of the fish that acted as serum donors in the passive immunization trial. NAI fish did not have parasite-specific IgM in serum, whereas SUR fish had survived an infection with *Enteromyxum leei* and had high levels of circulating parasite specific IgM. The diversity of the clonotypes sequenced for IgM **(B)** and IgT **(E)** was also evaluated using the Chao1 diversity index. The differential usage of VH-gene families for IgM **(C)** and IgT **(F)** was calculated with the assignment against zebrafish sequences. Bars represent the average number (mean ± SD) on unique sequences for *n* = 4 fish. Asterisks represent statistical differences between NAI and SUR groups at *P* < 0.05.

The next step we took was an attempt to characterize the type of Ig response elicited by the parasite. Thus, we studied the IgM and IgT repertoire in the GSB that acted as SUR and NAI serum donors for the passive immunization trial. By *in silico* analysis using immunoglobulin conserved domains, up to 46 regions showing homology with V genes were identified along the GSB genome (Supplementary Data S1). Following this identification, seven forward primers were designed in V regions which in combination with reverse primers located in IgM or IgT constant regions allowed for the first time the amplification of a global GSB repertoire (Supplementary Figure S2). Using Illumina MiSeq (2 × 300), an average of 2.2 million paired end reads per sample were obtained and assembled. Using the isotype specific primers as a barcode, 72.4 and 15.9% of the sequences were cataloged as IgM and IgT, respectively. Due to the absence of GSB reference sequences for VDJ genes, IMGT/HighV-QUEST analysis was performed using zebrafish as a reference. This analysis resulted in the identification of productive sequences as well as their CDR3, and served to assign VDJ genes and unique clonotypes according to homology to zebrafish, which allowed the comparison between experimental groups. After analysis, 93.2% of the IgM sequences and 84.7% of the IgT sequences were classified as productive (Supplementary Table S2). Analysis with immunarch revealed that the number of unique clonotypes was significantly higher for both isotypes in immunized (SUR) fish than in naïve (NAI) individuals (Figures 5A,D). The clonotype diversity (estimated with the Chao1 index) was also always significantly higher in SUR animals than in NAI fish (Figures 5B,E). Sequences classified as similar to IGHV5 and IGHV10 zebrafish subgroups were the most expressed in both isotypes. IGHV10 like sequences were significantly overexpressed in IgT, whereas IGHV5 like sequences were overrepresented in both isotypes in SUR fish (Figures 5C,F).

**FIGURE 6:**
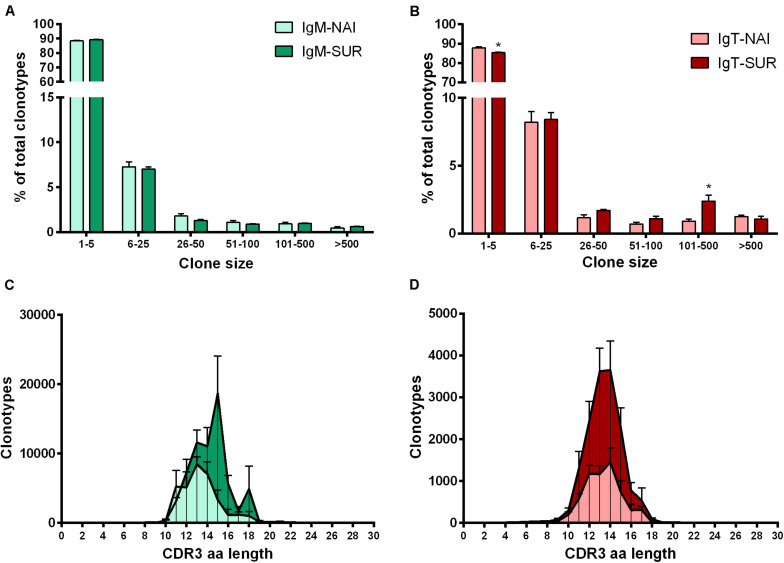
Clonal size distribution of IgM **(A)** and IgT **(B)** clonotypes in naïve (NAI, *n* = 4) fish and fish that acquired resistance to *Enteromyxum leei* (SUR, *n* = 4). Bars show the average percentage (mean + SD) of clonotypes observed *n* times in the datasets. Asterisks represent statistical differences between NAI and SUR groups at *P* < 0.05. CDR3 length distributions for all detected IgM **(C)** and IgT **(D)** clonotypes. The graphs represent the average number of clonotypes of a given amino acid (aa) length (+SD) for SUR and NAI fish. Kolmogorov–Smirnoff tests reported that none of the curves followed a bell-shaped Gaussian distribution (*P* > 0.05).

**FIGURE 7:**
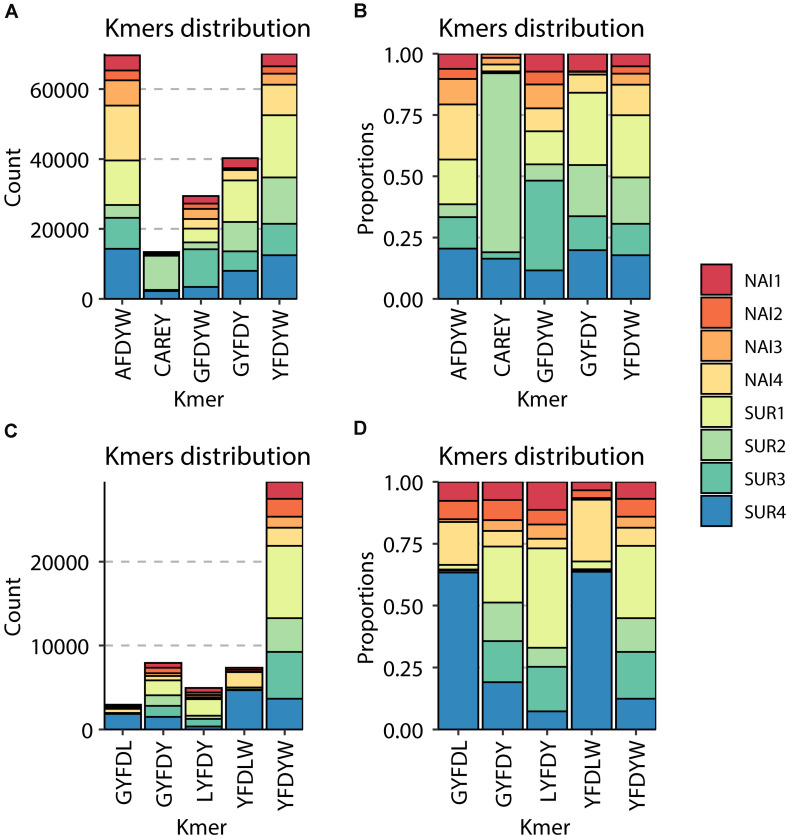
Graphical representation of the most abundant amino acid 5-mers in the CDR3 sequences of IgM **(A,B)** and IgT **(C,D)**. For each of the naïve (NAI, *n* = 4) and parasite resistant (SUR, *n* = 4) fish. The total count **(A,C)** and the relative proportion in the eight samples **(B,D)** of each of the most abundant 5-mers are represented.

Around 90% (IgM) and 85% (IgT) of the different clonotypes were found one to five times, indicating a broad diversity of Ig repertoire in GSB intestines. Interestingly, the percentage of IgT clonotypes found one to five times decreased in SUR animals, whereas IgT clonotypes, which were represented more than 100 times, increased in SUR fish. These results demonstrate a higher frequency of expanded IgT clonotypes in SUR fish, indicating a clear response. No significant changes were found for IgM (Figures 6A,B). When analyzing the CDR3 amino acids length of the different clonotypes, a shift to longer CDR3 lengths was observed for IgM, but no clear differences appeared in IgT distribution (Figures 6C,D). None of the CDR3 length histograms fitted the expected Gaussian bell distribution normally expected for naïve animals (Kolmogorov-Smirnov normality test *P* > 0.05). Analysis of the five most frequent k-mers (*k* = 5) from the CDR3 sequences revealed that the k-mer YFDYW is very frequent in both, IgM and IgT sequences, with a frequency increase in SUR individuals. The other detected k-mers were not common for both isotypes (Figure 7).

## Discussion

In mammals, passive immunization represents a prophylactic strategy when vaccination is not possible, such as in immunosuppressed or very young individuals. Also, in the case of certain cancers or emerging human diseases, when vaccines or treatments have not yet been developed (44, 45). In fish, passive transfer of spAbs by injection has been successful against some infectious diseases, including parasites (4, 5, 7). Opposite to other animal production sectors, aquaculture animals live in the aquatic environment, which hinders individual treatment approaches. Thus, passive immunization through injection is not the most feasible strategy for fish. However, research is nowadays being conducted on encapsulated antibodies to be delivered in-feed (46), as it was previously described for pigs (47). In any case, experimental injection trials, such as the one presented in this manuscript, allow to test whether spAbs could be effective for treating a particular infection and can serve as a base to develop future control strategies such as vaccination or in-feed approaches (8).

In passive immunization trials two main considerations are important: the time of the injected antibodies to reach the bloodstream, and the half-life of these antibodies in circulation (8), which will indicate the time-frame of protection. In salmonids, i.c. injected allogeneic IgM against vibriosis were detected in serum 10 min post-injection and the uptake was complete after 8 h. These antibodies remained protective for up to 60 days (48, 49). In carp, the half-life of allogeneic IgM was much shorter, 22.5 days (50). It is clear that the half-life of serum antibodies varies depending on the species, but teleost Igs are assumed to remain in circulation from 12 to 16 days still retaining the antigen binding efficiency (9). In the current study, we performed a preliminary test that revealed that i.c. injected rabbit IgG could be detected in GSB serum from 1 h to 9 days post-injection in high concentrations. However, IgG besides being xenogeneic, which could elicit a host response against the injected molecules, is also a monomer, so faster absorption times could be expected. In any case, these results, together with literature data, allowed us to plan the injection timings. Fish were challenged with the parasite 24 h after serum injection, which is a safe window to ensure complete uptake of the antibodies. A second injection was performed 9 dpc to ensure that injected antibodies remained in circulation during the whole challenge period (23 days).

The current passive immunization trial did not show significant protection of GSB against *E*. *leei*. However, the results indicate that injection with parasite-spAbs delayed the initial propagation of the parasite in naïve GSB. The group that received serum with parasite-spAbs (SUR) showed minor signs of infection, particularly in terms of SGR and intensity of infection than fish injected with serum without spAbs or PBS. *E*. *leei* invades and proliferates in the intestinal epithelium, causing anorexia, impaired nutrient absorption and weight loss, leading to emaciation (25). Decreased CF and SGR have been linked to *E*. *leei* infection in several studies (25, 51, 52). This effect was evident in the PBS and NAI-injected groups, showing a higher prevalence of infection. SUR-injected fish also were infected, reaching 55% of prevalence of infection 100 dpc. However, these animals showed less disease signs than the other two challenged groups, in coincidence with lower prevalence and progression of the infection. *E*. *leei* starts invading the posterior part of the intestine, slowly progressing to the anterior and finally to the middle segment of the intestine (25, 53). Hence, the number of intestinal segments parasitized serves as a measure of the progression of the infection and provides a hint of the time a fish has been parasitized. In fact, the number of intestinal segments parasitized was significantly correlated (negatively) with disease signs such as CF (34). In the same study, intensity of infection, measured as Ct values of the parasite 18S rRNA, was significantly correlated with CF, SGR and extension of the infection. Lower Ct values indicate more copies of parasite 18S rRNA and coincided with stronger disease signs and more intestinal segments parasitized (34). The current results are in agreement with this previous observation.

*Enteromyxum leei* induces a massive hyperplasia of the intestinal lamina propria-submucosa due to recruitment and proliferation of heterogeneous leukocytes (54). IgM^+^ cells massively proliferate in the submucosa and within the epithelium (27), whereas Zap70^+^ T cells aggregate mainly at the base of the epithelium (28). This inflammatory response, together with enterocyte apoptosis and necrosis, hamper intestinal barrier integrity producing nutrient malabsorption and osmotic intestinal failure (31). As expected, these signs were also observed in the current experiment, where challenged fish showed an inflammatory response in the submucosa with abundant Zap70^+^ T cells at the base of the epithelium and infiltrated in it. Nutrient absorption impairment was also reflected in the reduction of lipid and glycogen deposits in the liver, which were probably used as an energy source to compensate the low energy intake. Again, fish injected with parasite-spAbs, even though being infected, showed lower signs than the other groups, probably denoting a later onset of the disease.

Antibodies have already been described as main actors in GSB immune response against *E*. *leei* (29), and in other fish-myxozoan models (55–59). The presence of high levels of circulating parasite-specific IgM in GSB that survived an infection with *E*. *leei* has been linked to their resistance to re-infection (29). *E*. *leei* induces IgM and IgT gene expression both at local (intestine) and systemic levels (head kidney), with a potent local effect. This increased gene expression has been correlated with increased serum levels of parasite-specific IgM (29, 31). Regretfully, no information is available on levels of parasite-specific IgT or whether these spAbs can be found in circulation, as IgT has been described mainly as a mucosal Ig (20). B cell responses against *E*. *leei* have been detected from 40 dpc onward (26, 27, 29, 35), and this response is temperature dependent (34, 60). Delayed B cell responses against myxozoan parasites have already been described, with spAbs being detectable only after 6–8 weeks (61). In the current challenge, we did not observe major differences on circulating IgM and IgT among injected groups. The higher presence of total circulating IgT in the PBS injected group could be related to a higher reaction to the antigen, as this group was the one showing the higher prevalence and progression of the infection. However, the mechanism behind this and the specificity of the antibodies should be checked in future studies. Fish injected with specific-antibodies showed an increase in total IgM, but this increase was not reflected as a parasite-specific response, therefore, this difference remains to be explained. In any case, as SUR injected fish seem to have been protected in the first weeks of infection, a lower amount of parasite-spAbs would support the latter onset of the disease. However, all groups challenged with the parasite showed an increase in parasite-specific IgM in serum after 100 days, with no difference among groups. The overall low antibody response detected in the current experiment could be attributed to the low temperature as previously described (34).

The current results suggest that the injected antibodies may be acting at the initial steps of parasite invasion probably by blocking the parasite host-specific receptors (opsonization) and/or promoting phagocyte activity (62, 63). However, probably due to the long-term nature of the infection, the injected antibodies were not enough against the parasite pressure. Similar results have been found in other fish slow-progressing parasites models. Passively immunized rainbow trout showed a lower, although not significant, abundance of the monogenean *Gyrodactylus derjavini* one month after infection (6). Also in rainbow trout, passive immunization against the microsporidian parasite *Loma salmonae* was not enough to protect the fish against infection, but delayed its arrival at the heart by one week (64). In our experiment, fish were challenged with parasite containing effluent water for 23 days and then each group was separated to tanks with clean water to avoid excessive parasite pressure. Nonetheless, cohabitation is also an effective infection route for *E*. *leei* (34). Thus, even with one animal getting infected during the challenge phase, the disease can still be transmitted to the cohabitants at later time points, when passively transferred antibodies are no longer viable. Future experiments using repeated injections of serum throughout the whole period, injecting higher amounts of immune serum, and/or separating the fish individually after the challenge phase, will help to determine the exact degree of protection that can be acquired by passive immunization against *E*. *leei*.

The current results support the hypothesis from previous studies that a spAb response is key against enteromyxosis in GSB (29). Thus, we further analyzed the antibody response being elicited in the animals that acted as serum donors for the passive immunization trial. These animals had high levels of circulating parasite-specific IgM and were resistant to re-infection. The repertoire analysis was performed on the anterior intestine, as previous studies revealed that in this particular organ IgM and IgT transcript expression increased upon re-exposure to the parasite in resistant fish (29). The CDR3 of the VH domain was targeted because this region is the key determinant of specificity in antigen recognition in antibodies and the T cell receptor, whereas CDR1 and CDR2 sequences are much more cross-reactive (65).

A strong and specific response against a pathogen would induce a decrease in the clonotype diversity in favor of the expansion of a specific pathogen-specific subset. This phenomenon has been described in fish in some viral infections (17). Parasites, however, are much more complex organisms, in particular metazoans with different life stages like myxozoans. Parasites can harbor vast amounts of antigenic peptides and some even demonstrated specific strategies to avoid the host’s antibody responses. For instance, some parasites are known to induce hypergammaglobulinemia, resulting in a polyclonal expansion with significantly increased antibody titers but a limited proportion of parasite-spAbs. This has been reported for human and fish protozoan parasites, such as *Trypanosoma cruzi* (66) or *Trypanoplasma borreli* (67), but also in some myxozoan infections caused by *Sphaerospora molnari* (68) or *Tetracapsuloides bryosalmonae* (23). The current results, showing a significant increase in clonotype diversity for both IgM and IgT, together with previous findings on this host-parasite model (27, 35), seem to indicate a similar strategy for *E*. *leei*. Close to 90% of IgT and IgM clonotypes were found 1–5 times, indicating a high degree of repertoire diversity in GSB anterior intestine. This high clonotype diversity, with very few clonotypes expressed more than 50 times, has been described in naïve rainbow trout spleen and, upon viral (17) or parasitic (23) infection, having certain clonotypes a high frequency, and indicating a clear response against the pathogens. The results of the current study deal with the Ig repertoire of intestinal B cells, thus the pattern is expected to be different from that found in spleen. Intestine is a major mucosal tissue that is in close contact with the environment and harbors a very diverse community of symbiont microorganisms, the microbiota. Hence, intestinal cells are constantly exposed to a myriad of antigens. This probably explains the different patterns found in naïve animals in our study with more abundant high frequency clonotypes and non-Gaussian CDR3 length distributions. The CDR3 length distribution analysis is an estimate of the overall diversity and deviations from a bell-shaped Gaussian distribution are indicative of clonal expansions (69). The different distribution of IgM CDR3 lengths, and the significantly more abundant IgT clonotypes with frequencies of 101–500 in SUR animals indicate that *E*. *leei* is inducing a clear response that involves both isotypes but probably through different mechanisms. A complete characterization of a GSB specific reference germline throughout the definition of the GSB IgH locus following the identification of all segments encoding V, D and J genes will allow further deep analyses of this response at gene level. Thus, the definition of a GSB specific germline will allow a better dissection of clonotypes specifically selected during the response to myxozoans as well as will allow to further explore somatic hypermutation patterns in selected clonotypes.

To conclude, *E*. *leei* induces a differential IgM and IgT response in GSB characterized by a polyclonal expansion of diverse Ig subsets. This could be part of an immune-evasion strategy elicited by the parasite aiming to dilute parasite-spAbs by increasing the proportion of irrelevant antibodies, as has been described in similar host-parasite models (23, 68). However, increased levels of parasite-spAbs (IgM) have been detected in the serum of GSB when challenged with the parasite, and the presence of high levels of these antibodies has been related with acquired resistance. In addition, the current results show that passive transfer of parasite-spAbs can delay the establishment of the infection and the appearance of disease signs. Taken together, these findings indicate that, regardless of the probable immune-evasion strategy of the parasite, antibodies are key molecules against enteromyxosis in GSB. Future studies should be conducted to further evaluate the role of specific IgT, a specialized mucosal immunoglobulin, in this model. Also, more in depth studies on the protective antibody response will be key to develop effective treatments and prophylactic methods against *E. leei*.

## Data Availability Statement

The datasets presented in this study can be found in online repositories. The names of the repository/repositories and accession number(s) can be found here: https://www.ncbi.nlm.nih.gov/, PRJNA644800.

## Ethics Statement

The animal study was reviewed and approved by Ethics and Animal Welfare Committee of IATS, CSIC and Generalitat Valenciana.

## Author Contributions

AP-S, IE, RP, and MP performed the fish trials and samplings. RP performed the molecular parasite diagnosis. IE and AS-B performed the histological evaluations and parasite diagnosis. AP-S and IE performed the immunohistochemistry and biochemical studies. AP-S and MP performed the repertoire PCR experiments. PP, CT, and MP designed the repertoire study and performed data analysis and interpretation. AP-S and MP wrote the original draft of the manuscript. AS-B and CT performed the funding acquisition. All authors contributed to the further editing and revisions.

## Conflict of Interest

The authors declare that the research was conducted in the absence of any commercial or financial relationships that could be construed as a potential conflict of interest.

## Footnotes

1 https://www.genome.jp/tools-bin/clustalw

2 http://hannonlab.cshl.edu/fastx_toolkit/
