# Supplementary material for: Passive Immunization Delays Disease Outcome in Gilthead Sea Bream Infected With Enteromyxum leei (Myxozoa), Despite the Moderate Changes in IgM and IgT Repertoire
Source: Front Immunol. 2020 Sep 11;11:581361. doi: 10.3389/fimmu.2020.581361 (PMC7516018; doi:10.3389/fimmu.2020.581361)
Supplement: Supplementary file 1 [file Table_1.PDF]

Supplementary Table S1: Primers used for repertoire analysis in gilthead sea bream.

**Primer name**	**Sequence**	**VH family***
IgHV1-1-FW	TCAGACAGGCTCCTGGAAAAG	ighv6-1, ighv6-2, ighv10-1
IgHV1-2-FW	AAGGAAAAGGACTGGAGTGG	ighv3-1, ighv11-2
IgHV4-FW	AGGGAAAGCTCTGGAGTGGAT	ighv2-2
IgHV8-FW	CTGGATCAGACAACCTGCAG	ighv4-1, ighv4-2, ighv4-5
IgHV6-FW	TGGATCAGACAGCCTGCAG	ighv5-7
IgHV12-1-FW	TGAAGGAAAACCAATGGACTGGAT	ighv5-1, ighv5-5
IgHV12-2-FW	AGACCTTTCCAGCAACACAGT	ighv5-3
**Primer name**	**Sequence**	**Isotype (domain)**
IgHCm2-2-RV	GAACGTAACGGCTGGTGGATT	IgM (Cμ2)
IgHCt1-RV	AACCAAAGGAAACAGAGTCGG	IgT (Cτ1)

*Provisional names based on domain prediction and similarity with zebrafish VH genes.
