# Supplementary material for: Passive Immunization Delays Disease Outcome in Gilthead Sea Bream Infected With Enteromyxum leei (Myxozoa), Despite the Moderate Changes in IgM and IgT Repertoire
Source: Front Immunol. 2020 Sep 11;11:581361. doi: 10.3389/fimmu.2020.581361 (PMC7516018; doi:10.3389/fimmu.2020.581361)
Supplement: Supplementary file 2 [file Table_2.PDF]

Supplementary Table S2: Summary of sequence analysis for the gilthead sea bream repertoire study. The total sequence column represents the sequences catalogued using the isotype specific primers as barcodes. These correspond to the input datasets for IMGT/HighV-QUEST analysis and were further analysed using VDJtools software.

Isotype	Sample	Total Sequences[1]	Productive[2]	%	Unique Productive[3]
IgM	NAI1	1212502	1111726	91.69	27893
	NAI2	1085478	946860	87.23	18975
	NAI3	1364383	1239361	90.84	31576
	NAI4	1198061	1102736	92.04	54676
	SUR1	1945213	1860441	95.64	88564
	SUR2	1282689	1245484	97.10	42655
	SUR3	1346150	1278501	94.97	60507
	SUR4	1279122	1225746	95.83	68045
IgT	NAI1	306390	256978	83.87	5088
	NAI2	425306	322885	75.92	5182
	NAI3	222954	140843	63.17	3710
	NAI4	261887	223047	85.17	10054
	SUR1	695011	662240	95.28	19238
	SUR2	203544	189540	93.12	8905
	SUR3	334300	310810	92.97	12328
	SUR4	387898	341613	88.07	20453

[1] Total sequences used as input for IMGT-HighVQuest after quality filtering. [2] Total productive sequences according to IMGT-HighVQuest. [3] Functional clonotypes according to parse from VDJtools.
