# Supplementary material for: Passive Immunization Delays Disease Outcome in Gilthead Sea Bream Infected With Enteromyxum leei (Myxozoa), Despite the Moderate Changes in IgM and IgT Repertoire
Source: Front Immunol. 2020 Sep 11;11:581361. doi: 10.3389/fimmu.2020.581361 (PMC7516018; doi:10.3389/fimmu.2020.581361)
Supplement: Supplementary file 3 [file Data_Sheet_1.PDF]

**Supplementary Data S1**: IGHV candidate sequences identified from the gilthead sea bream genome. The nomenclature of each gene is provisional and was performed by identifying the most similar IGHV gene in zebrafish.

>ighv2-2like.1

GAATGTCATTTAAATCACGCTTGTTGTCTGTGGAGGTTTTTGTGCAGCTGCTCCACTGTCTTCAGTCACTGTGTCTC
TCGAGCACAGAAGTAAACAGCAGAATCTTCTGCTGTCAGGCTCCTGATCTCGAGGTACTGAGTGCTGCTGGACACGT
CTTCAGTCATGAGGAAACCACTTTGAAAGGAGCTGGCATAGATTGCAGAGTTTTTTCCAGTATCCATCCTCCCAATC
CACTCCAGAGCTTTCCCTGGCTTCTGTCGTATCCAGTGAATATTGCGGCTCGTCATGCTATAACCAGATATGATACA
TGACATCTTCACTGTCTCTCCAGGTGTTTTCACCTCAGAGGGAGACTGGTCCAGTTTG

>ighv2-2like.2

TACAACACGTCGACACCCACATTTTCGTGTCAATACATTTTTTTTAATCGTTGTAGCCCTAGTCTCTACGAGCCCAG
AAGTAAACAGCAGAATCTTCTGCTGTCAGGCTCCTGATCTCGAGGTACTGAGTGCCGCTGGGCACGTCTTCAGTCAT
GAGGAAACCACTTTGAAAGGAGCTGGCATAGATTGCAGAGTTTTTTCCAGTATCCATCCTCCCAATCCACTCCAGAG
CTTTCCCTGGCTTCTGTCGTATCCAGTTAATAGCGTAGCTCGTCATGCTATAACCAGATATGATACATGACATCTTC
ACTGTCTCTCCGGGTGTTTTCACCTCAGAGGGAGACTGGTCCAGTTTG

>ighv2-2like.3

GATGAAGCCCACTGGGGAGGTTTTTGTGCAGCTGCTCCACTGTCTTCAGTCACTGTGTCTCTACGAGCACAGAAGTA
AACAGCAGAATCTTCTGCTGTCAGGCTCCTGATCTCGAGGTACTGAGTGCTGCTGGACACGTCTTCAGTCATGAGGA
AACCACTTTGAAAGGAGCTGGCATAGCTAGCAGAGTTTGAACCTGCGTTCATCCTCCCAATCCACTCCAGAGCTTTC
CCTGGCTTTTGTCGTATCCAGTGAATAAAGTAGTCTGTCATGTCAAACCCAGATATGATACATGACATCTTCGCTGT
CTCTCCGGGTGTTTTCACCTCAGAGGGAGACTGGTCCAGTTTG

>ighv2-2like.4

GAAGGTCACATGTTTGTTGTCTGTAGAGGTTTTTGTGCAGCTGCTCCACTGTCTTCAGTCACTGTGTCTCTGCGAGC
ACAGAAGTAAACAGCAGAATCTTGTGCTGTCAGGCTCCTGATCTCGAGGTACTGAGTGCTGCTGGACACATCTTCAG
TCATGAGGGAACCACTTTGAAAGGAGCTGGCATAGCTAGCAGAGTTTGAACCTGTGCTCATCCACCCAATCCACTCC
AGAGCTTCCCCTGGTTTCTGTCGTATCCAGACCATATTAGCACTCTTCATGCTATAACCAGATATGATACATGACAT
CTTCACTGTCTCTCCAGGTCTTTTCACCTCAGAGGGAGACTGGTCAAGTTTG

>ighv2-2like.5

GAAGATCACATGTTTGTTGTCTGTGGAGGTTTTTGTGCAGCTGCTCCACTGTCTTCAGTCACTGTGTCTCTCGAGCA
CAGAAGTAAACAGCAGAATCTTCTGCTGTCAGGCTCCTGATCTCGAGGTACTGAGTGCTGCTGGACACGTCTTCAGT
CATGAGGAAACCACTTTGAAAGGAGCTGGCATAGATAGCAGAGTTTCAACCTGTGTTCATCCACCCAATCCACTCCA
GAGCTTTCCCTGGCTTCTGTCGTATCCAGACCATATTGTAGCTCATCATGCTATAACCAGATATGACACATGACATC
TTCACTGTCTCTCCAGGTGTTTTCACGTCAGAGGGAGACTGGTCCAGTTTG

>ighv2-2like.6

GAAGATCACATGTTTGTTGTCTGTGGAGGTTTTTGTGCAGCTGCTCCACTGTCTTTAGTCACTGTGTCTCTCGAGCA
CAGAAGTAAACAGCAGAATCTTCTGCTGTCAGGCTCCTGATCTCGAGGTACTGAGTGCTGCTGGACACGTCTTCAGT
CATGAGGAAACCACTTTGAAAGGAGCTGGCATAGATAGCAGAGTTTGAACCTGCGTTCATCCTCCCAATCCACTCCA
GAGCTTTCCCTGGATTCTGTTGTATCCAGTAAATATTGTAGCTCGTCATTCTATGACCAGATATGATACATGACATC
TTCACTGTCTCTCCAGTAGGGCTGGGCGATTATGCTAAAAATAATAATCAAGATTA

>ighv2-2like.7

GAAGATCACATGTTTGTTGTCTGGGGAGGTTTTTGTGCAGCTGCTCCACTGTCTTCAGTCACTGTGTCTCTCGAGCA
CAGAAGTAAACAGCAGAATCTTCTGCTGTCAGGCTCCTGATCTCGAGGTACTGAGTGCTGCTGGACACGTCTTCAGT
CATGAGGAAACCACTTTGAAAGGAGCTGGCATAGCTAGCAGAGTTGTAACCTGTGTTCATCCACCCTATCCACTCCA

GAGCTTTCCCTGGCTTCTGTCGTATCCAGTGAATATTGTAGTCTGTCATGTCAAACCCAGATATGATACATGACATC
TTCACTGTCTCTCCAGGTGTTTTCACCTCAGAGGGAGACTGGTCCAGTTTG

>ighv2-2like.8

GAAGATCACATGTTTGTTGTCTGGGGAGGTTTTTGTGCAGCTGCTCCACTGTCTTCAGTCACTGTGTCTCTCGAGCA
CAGAAGTAAACAGCAGAATCTTCTGCTGTCAGGCTCCTGATCTCGAGGTACTGAGTGCTGCTGGACACGTCTTCAGT
CATGAGGAAACCACTTTGAAAGGAGCTGGCATAGCTAGCAGAGTTGTAACCTGTGTTCATCCACCCTATCCACTCCA
GAGCTTTCCCTGGCTTCTGTCGTATCCAGTGAATATTGTAGTCTGTCATGTCAAACCCAGATATGATACATGACATC
TTCACTGTCTCTCCAGGTGTTTTCACCTCAGAGGGAGACTGGTCCAGTTGATCTC

>ighv3-2like.1

GAAAGTAAAGTTTTGACTTGTTTTGAAATGTTGAGTTTTTGTACAGCGTTGCTCCTTCCTGTGTCACTGTGTCTCTC
GAGCACAATAATAAACAGCAGAATCTTCAGTCTTCAGACTGTTCATCTGCAGATACACCTGCTGTCTGCTGTTGTCT
CTGGAGATGGTAAACCGGCCTTTGACTGTTTCAGAGTAGTAAGTGCTGCCACCACTAGGATAGATCTCAGCAAGCCA
CTCCAGTCCTTTTCCAGGAGCCTGTCTGATCCAGTGCATAGCATAGTCACTGAATGTGAATCCAGAGGCTGTACAGG
TCAATCTGTGAGATTCTCCAGGCCTTTTAACCACTGGTTCAGATTCTGTCAGAGTCTG

>ighv3-2like.2

AACTGTTATTATTTTTCAGGCTCTTTCAGCCCAGTAAAGATGAGTTCTGTAGGTTTTTGTACAGCTGCTCAACAATT
CCAGTCACTGTGGCTCTCGAGCACAATAATAAACAGCAGAATCTTCAGTCTTCAGACTGTTCATCTGCAGATACACC
TGCTGTCTGCTGTTGTCTCTGGAGATGGTAAACCGGCCTTTGACTGACTCAGAGTAGTAGATGTAGCTACTGCCATC
ACTACTGATATAAGCAACCCACTCCAGTCCTTTTCCAGGAGCCTGTCTGATCCAGTTCATAGGAGAGCTGCTGAATG
TGAATCCAGAGGCTGTACAGGTCAATCTGTGAGATTCTCCAGGCCTTTTAACCACTGGTTCAGATTCTGTCAGAGTC
TG

>ighv3-2like.3

CAGGTGCAAAACTGAAAGTTTTTGGCGGCCCTCTAGGTGGCGCTTGCGGCCCAAGCTTGGGCCGCGGCCCTATGGTT
AAGAATCACTGGTCTAAATCAAGTGAAAATACGCAGAAGAAGACACTACAAGTTGTTTTTACATGTTGAGTTTTTGT
TAAGGGTTGCTCCTTCCTGTGTTGCTGTGGCTGTCGAGCACAATAATAAACAGCAGAATCTTTAGTCTTCAGACTTC
AGACTTCCTTTTCCAGGAGCCTGTCTGATCCAGTTCATCTCGTAGCTGCTGAATGTGAATCCAGAGGCTGTACAGGT
CAATCTGTGAGATTCTCCAGGCCTTTTAACCACTGGTTCAGATTCTGTCAGAGTCTG

>ighv3-2like.4

CCCAGTAAAGATGAATTCTGTAGGTTTCTGTACAGCTGCTCAACCAACTCCAGTCACTGTGGCTGTCGAGCACAATA
ATAAACAGCAGAATCTTCAGTCTTCAGACTGTTCATCTGCAGATACACCTGCTGTCTGCTGTTGTCTCTGGAGATGG
TAAACCGGCCTTTCACTGACTCAGAGTAGTAGATGTAACTACTGCCACTGCTGATATACGCAACCCACTCCAGTCCT
TTTCCAGCAGCCTGTCTGATCCAAGCAGTATTGATACTGCCACCAAACCCTGAATATGTGCAGGTCAAACTGTGGGA
TTCTCCAGGTCTTTTAACCGCAGGTTCAGACTCAGTTAGTGTTTG

>ighv3-2like.5

CCCAGTAAAGATGAATTCTGTAGGTTTCTGTACAGCTGCTCAACCAACTCCAGTCACTGTGGCTGTCGAGCACAATA
ATAAACAGCAGAATCTTCAGTCTTCAGACTGTTCATCTGCAGATACACCTGCTGTCTGCTGTTGTCTCTGGAGATGG
TAAACCGGCCTTTCACTGACTCAGAGTAGTAGATGTAACTACTGCCACTGCTGATATACGCAACCCACTCCAGTCCT
TTTCCAGCAGCCTGTCTGATCCAAGCAGTATTGATACTGCCACCAAACCCTGAATATGTGCAGGTCAAACTGTGGGA
TTCTCCAGGTCTTTTAACCGCAGGTTCAGACTCAGTTAGTGTTTG

>ighv3-2like.6

ATGCTTCATGTTTTGGCTCCTTCAGCCCAGTAAAGATGAGTTCTGTAGGTTTTTGTACAGCTGCTCAACAACTCCAG
TCACTGTGGCTTTCGAGCACAATAATAAACAGCAGAATCTTCAGTCTTCAGACTGTTCATCTGCAGATACACCTGCT
GTCTGCTGTTGTCTCTGGAGATGGTAAACCGGCCTTTGACTGACTCAGAGTAGTATTGTTTGCTACCACTAGGTTGA

CTGACTAAAGCAACCCACTCCAGTCCTTTTCCAGGAGCCTGTCTGATCCAGTGCATCTCATAGTCACTGAATGTGAA
TCCAGAGGCTGTACAGGTCAATCTGTGAGATTCTCCAGGCCTTTTAACCACTGGTTCAGATTCTGTCAGAGTCTG

>ighv3-2like.7

ATGCTTCATGTTTTGGCTCCTTCAGCCCAGTAAAGATGAGTTCTGTAGGTTTTTGTACAGCTGCTCAACAACCCAGT
CACTGTGGCTTTCGAGCACAATAATAAACAGCAGAATCTTCAGTCTTCAGACTGTTCATCTGCAGATACACCTGCTG
TCTGCTGTTGTCTCTGGAGATGGTAAACCGGCCTTTGACTGACTCAGAGTAGTATTGTTTGCTACCACTAGGTTGAC
TGACTAAAGCAACCCACTCCAGTCCTTTTCCAGGAGCCTGTCTGATCCAGTGCATCTCATAGTCACTGAATGTGAAT
CCAGAGGCTGTACAGGTCAATCTGTGAGATTCTCCAGGCCTTTTAACCACTGGTTCAGATTCTGTCAGAGT

>ighv4-1like.1

GTAGGTCTTTGGAAACAAATGTTCTGTGGAGTTTTTGTAAAGCTTCTCTGCTTCCTTTAACCAGTGTGTGTGTTTGG
CACAGTAATACACAGCAGAGTCCTCTGGCTTTAAGTTGGACAGTCTCAGATACACCATGCTGTTGCTATCTTCTCTA
CTGATTTCTGCACGTCCTCGCACACTGCTGGCATAAGTGTTACTACTTGCATCAGTGAAGCCTTCCCCGATCCATTC
CTAGTCCTTTTCCTGCAGGTTGTCTGATCCAGCTCATAGTGCAGCAGCTGAATGTGAAGCCAGATCCCATGCAGGAC
AGACTGAGAGTCTCCCCAGGCTTTTTCACTACTGAACTGGAAGGAATGGACTCCA

>ighv4-2like.1

GTAGGTCTTTGGAAACAAATGTTCTGTGGAGTTTTTGTAAAGCTTCTCTGCTTCCTTTAACCAGTGTGTGTGTTTGG
CACAGTAATACACAGCAGAGTCCTCTGGCTTTAAGTTGGACAGTCTCAGATACACCATGCTGTTGCTATCTTCTCTA
CTGATTTCTGCACGTCCTCGCACACTGCTGGCATAAGTGTTACTACTGGAGCTGGAGTAGCCATTCCCGATCCATTC
CAGTCCTTTTCCTGCAGGTTGTCTGATCCAGCTCATAGTGCAGCAGCTGAATGTGAAGCCAGATCCCCTGCAGGACA
GACTGAGAGTCTCCCCAGGATTTTTCACTACTGAACTGGAAGGAATTGACTCCATACTCTG

>ighv4-2like.2

GTAGGTCTTTGGAAACAAATGTTCTGTGGAGTTTTTGGAAAGCTTCTCTGCTTCCTTTAACCAGTGTGTGTGTTTGG
CACAGTAATACACAGCAGAGTCCTCTGGCTTTAAGTTGGACAGTCTCAGATACACCATGCTGTTGCTATCATCTCTA
CTGATTTCTGCACGTCCTCGCACACTGCTGGCATAAGTGTTACTACTGGAGCTGGAGAAGCCAACCCCGATCCATTC
CAGTCCTTTTCCTGCAGGTTGTCTGATCCAGCTCATAGTGCAGCAGCTGAATGTGAAGCCAGATCCCATGCAGGACA
GACTGAGAGTCTCCCCAGGCTTTTTCGCTACTGAACTGGAAGGAATGGACTCCA

>ighv4-2like.3

GTAGGTCTTTGGAAACAAATGTTCTGTGGAGTTTTTGTAAAGCTTCTCTGCTTCCTTTAACCAGTGTGTGTGTTTGG
CACAGTAATACACAGCAGAGTCCTCTGGCTTTAAGTTGGACAGTCTCAGATACACCATGCTGTTGCTGTCATCTCTA
CTGATTTCTGCACGTCCTCGCACACTGCTGGCATATGTGTTTTTACTGGAGGTGGAGTAGCCAACCCCGATCCATTC
CAGCCATTTTACTGCAGGTTGTCTGATCCAGTGCATACTGCAGCAGCTGAATGTGAAGCCAGATCCCCTGCAGGACA
GACTGAGAGTCTCCCCAGGCTTTTTCACTACTGAACTGGAAGGAATGGACTCC

>ighv4-5like.1

GTAGGTCTTTGGAAACAAATGTTCTGTGGAGTTTTTGTAAAGCTTCTCTGCTTCCTTTAACCAGTGTGTGTGTTTGG
CACAGTAATACACAGCAGAGTCCTCTGGCTTTAAGTTGGACAGTCTCAGATACACCATGCTGTTGCTATCTTCTCTA
CTGATTTCTGCACGTCCTCGCACACTGCTGGCATAAGTGTTACTACTTGCATCAGTGAAGCCTTCCCCGATCCATTC
CTAGTCCTTTTCCTGCAGGTTGTCTGATCCAGCTCATAGTGCAGCAGCTGAATGTGAAGCCAGATTAATGTCAGGGT
TTTCCCTGCCATTATACGGCTTAGGCGTGGCGCCCAAGCGTTTTGCCTCCCGCCTAAGCAGG

>ighv5-1like.1

GTTGAGTCACATGGTCTCTGGGTATTTGTGCAGGTCTGTTGGTGGTTTGTATCACTGTGGGCTGACGTAGACAGTAA
TAAACAGCTGTGTCCTCAGGCTGCAGATTCTGTCCTGTTAGAGTCACTGTTCTAGCAGAAGTGTCTCTGCTGTAGCT
GAACTTGTTCTTCAGAGCATTATTTTGATAAAGGCCACCTCCACCCCATATGGAAAATCCAGTCCATTGGTTTTCCT

TCACACTTGTCTTCAATGTGTACACTGAATTAATTTCATTACTTGACTTTATCATGTACTTAAATTAAATGTACTGT
AATTTGTTTAAGAAAATGGATCACCCTTTATGAAGCCCAAGTATTTAAGTTGAAATTGAATTAA

>ighv5-3like.1

AATGCATTACTTTGTACAAGTGTTCAGGCACTGAGGGGTTTTTGTTCAGGTCTACTGATGGTTTGTGTTATTGTGGC
TCTCTGGCACAGTAATACACAGCAGTGTCTTCAGACTGCACATTATGTCCATTTAGAGTCACTGTGTTGCTGGAAAG
GTCTGTTGACTGTTATTATATCTGTAGAGTGAAAACTTGCCTGGAAATCACCTCTGCTTTACACATGATATTTTCAT
TTCATTACACACTCAACTGTAACATGCTCCTCCTGTGCTGACACACTGACACTGACTTCATTCACCTGAAATGAGAG
TCAAGAAGAAGAAACTTAGTACAACTTGGCTGAGTCTGCAACTGCAAAGGATATATGAAGGATATACACCGATCAG

>ighv5-3like.2

AATGCATTACTTTGTACAAGTGTTCAGGCACTGAGGGGTTTTTGTTCAGGTCTACTGATGGTTTGTGTTATTGTGGC
TCTCTGGCACAGTAATACACAGCAGTGTCTTCAGACTGCACATTATGTCCATTTAGAGTCACTGTGTTGCTGGAAAG
GTCTGTTGACTGTTATTATATCTGTAGAGTGAAAACTTGCCTGGAAATCACCTCTGCTTTACACATGATATTTTCAT
TTCATTACACACTCAACTGTAACATGCTCCTCCTGTGCTGACACACTGACACTGACTTCATTCACCTGAAATGAGAG
TCAAGAAGAAGAAACTTAGTACAACTTGGCTGAGTCTGCAACTGCAAAGGATATATGAAGGATATACACCGATCAG

>ighv5-5like.1

TACAATCTTGAAATTCAAGTTCAGATCCACATATATTGCAACACCACCACCCCCTTTGCTCTGTCTGTTTTAACACA
GTAATAAACAGCTGTGTCCTCAGGCTGCAGATTCTGTCCTGTCAGAGTCACTGTTCTAGCAGAAGTGTCTCTGCTGT
AGCTGAACTTGTTCTTCAGAGCAATATTTTGACAAAGGGTACCTCCACCCCACTGATGGGAAATCCAGTCCATTGGT
TTTCCTTCACACTGTCTGATCCAACCTGTTGCATAGCTGTCATCAGTCAAATAATAACCAGAGACCTGACAGGTGAT
GGTCAAAGACTGTCCAGGCTGCACAACCTTTGAGTCTGGCTGGATGAGATCAA

>ighv5-5like.2

AACTTTTGTGGTTGAGTCACATGGTCTCTGGGTATTTGTGCAGGTCTGTTGGTGGTTTGTATCACTGTGGGCCGACG
TACACAGTAATAAACAGCTGTGTCCTCAGGCTGCAGATTCTGTCCTGTTAGAGTCACTGTTCTAGAAGAAGTGTCTC
TGCTGTAGCTGAACTTGTTCTTCAGAGCATTATTTTGATAAAAGTTGCCTCCACCCCACTGATAAGAGATCCAGTCC
ATTGGTTTTCCTTCACACTGTCTGATCCAACCTATTGCATAGCTGCTGTCAGTCAAAGAATAACCAGAGACCTGACA
GGTGATGGTCAAAGACTGTCCAGGCTGCACAACCTTTGAGTCTGGCTGGATGAGATCAA

>ighv5-7like.1

GACAAGTGAATGTTTCCAGTGGAGGTAAATAGAGGAAGTAGTTTTTGTATGACTCTCCTGTTATTGTCTCTCACTGT
GGGTCTCTGGCACAGTAATACACAGCAGTGTCTTCAGACTGCACATTCTGTCCATTTAGAGTCAATGTTTTACTGGA
AGAGTCTAAATCGATACTGAATTTGTTCTTTAGGGAATCTTTGTAGTATGTACTGTATCCAACACGTGCACTCCCAA
TCCACTCCAGTCCTTTCCCTGCAGGCTGTCTGATCCAGTGTGTGCGATAGCTGCTAACAGAATAAGAGACCTGACAG
GTGATGGTCAGACGTTGACCTGGCTGCACAGTCACAGAGGCTGGCTGTATCAACTGTT

>ighv5-7like.2

TACTGAGACAACTGAATGTTTCCAGTGGTGGTAAATAGAGGAAGTACTTTTTGTAAGACTCTCCTGTTATTGTCTCA
CACTGTGTCTCTTCTGGCACAGTAATACACAGCAGTGTCTTCAGACTGCACATTCTGTCCATTTAGAGTCACTGTTT
TACTGGAAGAGTCTAAATCGATACTGAATTTGTTCTTGAGGGAATCTTTTACTTGTGTGTCATCACCCATCCACTCC
AGTCCTTTCCCTGCAGGCTGTCTGATCCAAGCTGTAGCATAGATGCTAACAGAATAAGAGACCTGACAGGTGATGGT
CAGACGTTGACCTGGCTGCACAGTCACAGAGGCTGGCTTGTCAACTGTTCA

>ighv5-7like.3

GGTGGTAAATAGAGGAAGTAGTTTTTGTATGACTCTCTTATTATTGTCTCTCACTGTGGGTCGTCTGGCACAGTAAT
ACACTGCAGTGTCTTCAGACTGCACATTCTGTCCATTTAGTGTCACTGTGTTGCTGGAAGAGTCTAAATTGACACTG
AACTTGTTCTTTAGTGAATCTTTGACGTCAGTGTCATCACTCATCCACTCCAGTCCTTTCCCTGCAGGCTGTCTGAT

CCAAGCTGTGAAGTCGCTGCTAACAGAATAAGAGACCTGACAGGTGATGGTTAGACGTTGACCTGGCTGCACAGTCA
CAGAGGCTGGCTGTGTCAACTGT

>ighv5-7like.4

GAATGTTTCCAGTGGTGGTAAATAGAGGAAGTAGTTTTTGTATGACTCTCCTGTTATTGTCTCTCACTGTGGCTCTC
TGGCACAGTAATACACAACAGTGTCTTCAGACTGCACATTCTGTCCATTCAGAGTCACTGTGTTGCTGGAAGAGTCT
AAGTTGATACTGAACTTGCTCTTTAGTGAATCTTTGAAGTATGAGCCTCCAGTATTTTTCATCCCAATCCACTCCAG
TCCTTTCCCTGCAGGCTGTCTGATCCAAGCTGTGTGGTAGCCAAGAAAATAAGAGACCTGACAGGTGATGGTCAGAC
GTTGACCTGGCTGCACAGTCACAGAGGCTGGCTGTGTCAACTGTTCA

>ighv5-7like.5

TCATGTTACAAGTGTTCAGGCACTGAGGGGTTTTTGTTCAGGTCTACTGATGGTTTGTGTTATTGTGGCTCTCTGGC
GCAGTAATACACAGCAGTGTCTTCAGACTGCACATTCTGTCCATTTAGAGTCACTGTGTTGCTGGAAGAGTCTAAAT
CAATACTGAACTTGTTCTTTAGTGAATCTTTGTAGTACGAGGCTCTAGTGAATTTGCTTCCAATCCACTCCAGTCCT
TTCCCTGCAGGCTGTCTGATCCAAGCTGTCCAGTAGCTGCTAACAGAATAAGAGACCTGACAGGTGAGTCAGACGTT
GACCTGGCTGCACAGTCACAGAGGCTGGCTGTGTCAACTGTTCA

>ighv5-7like.6

GGTGGTAAATAGAGGAAGTAGTTTTTGTATGACTCTCCTATTATTCTCTCTCACTGTGGTTCGTCTGGCACAGTAAT
TCACAGCAGTGTCTTCAGACTGCACATTCTGTCCATTTAGAGTCACTGTGTTACTGGAAGAGTCTACATTGACACTG
AACTTGTTCTTAAGTGCATCTTTGACGTTAGTGTCATCACTCATCCACTCCAGTCCTTTCCCTGCAGGCTGTCTGAT
CCAAGTTGTGTAGTAGTCACTAACAGAATAAGAGACCTGACAGGCAGTGGTGTGCACAGGTAGACACTAGGTGGTGC
TAAAGCACCTGCCCTTTGCCCTCTGGACCAAGCAAAAGCCCTTTTGAAAGTTTT

>ighv6-1like.1

GTGACTTTGTGCAGCACAACTCAGTTAGAAGAGGAAGTAGTTTTTGTATGACTCTCCTGTTATTGTCTCTCACTGTG
TCTCTCTGGCACAGTAATACACTGCTGTGTCCTCAGTCATCTGTAGGTAGACTTTACTGCTGGAGTCATCTCTGGAG
ATGGTGAATCTACCCTTGACTGACTCGGAGTAATAGATAGGAGTACTATCTGTGTCTATAAAAGCAATCCACTTTTC
CAGAAGCCTGTCTGATCCAGTTCCAGACTGAGCCTTCAAAGTCAAATCCAGATGTTGTGCAGGTGAGTCTGTGGGAC
TCTCCGGGTCTTTTAACTGCTGGTTCAGACTCTGTTATGGTCC

>ighv6-2like.1

CATATTACATTTTCCACCAGTGTAGTAAACAGATGAAGTAGTTTTTGTATGACTCTCCTGTTATTGTCTCTCACTGT
GTCTCTCTGGCACAGTAATACACTGCTGTGTCCTCAGTCTTCAGACTGTTCATCTGTAGGTAGACTTTACTGCTGGA
GTCATCTCTGGAGATGGTGAATCTTCCCTTGACTGACTGGGTAATATAGATGCTAGTGCTGCCTGTGTGTATGAAAG
CGATCCACTCCAGTCCTTTTCCAGGAGCCCCAGAGTAGAATTTCCTGCCATTATCATATTCAACCCAGGCAATCCAC
TCCAGTCCTTTTCCAGGAGCCTGTCTGACCCAGTTCATATCATAGCTGCTGAATGTGAATCCAGAGGTTGTACAGGT
CAATCTGTGAGATTCTCCAGGCCTTTTAACCACTGGTTCAGATTCTTTCAGAGTCTG

>ighv10-1like.1

GGGCTCCCTCAACTCAGTAAAGATGAGTACTGTAGGTTTTTGTACAGCTGCTCAACAACTCCAGTCACTGTGTCTCT
CGAGCACAATAATAAACAGCAGAATCTTCAGTCTTCAGACTGTTCATCTGCAGATACACCTGCTGTCTGCTGTTGTC
TCTGGAGATGGTAAACCGGCCTTTGACTGACTCAGAGTAGTATTGGGTGCTACCACTGCTATAAATCCAGGCAACCC
ACTCCAGTCCTTTTCCAGGAGCCTGTCTGATCCAGTTCATATTGTAGCTGCTGAATGTGAATCCAGAGGCTGTACAG
GTCAATCTGTGAGATTCTCCAGGCCTTTTAACCACTGGTTCAGATTCTGTCAAAGTCTG

>ighv10-1like.2

GTCAGACAGCAAAGATGAGTACTGTAGGTTTTTGTACAGCTGCTCAACCAACTCCAGTCACTGTGGCTCTCGAGCAC
AATAATAAACAGCAGAATCTTCAGTCTTCAGACTGTTATCTGCAGATACACCTGCTGTCTGCTGTTGTCTCTGGAGA
TGGTAAACCGGCCTTTGACTGACTCAGAGTAGTAGATGCTGTTACTGTCATATCTGATAGCAGCAACCCACTCCAGT

CCTTTTCCAGGAGCCTGTCTGACCCAGTTCATCCAGTAGTCACTGAATGTGAATCCAGAGGCTGTACAGGTCAATCT
CTGAGATTCTCCAGGCCTTTTAACCACTGGTTCAGATTCGGTCAGAGTCTG

>ighv10-1like.3

AATACTTCATACTTGGCTCCATCAGTCCAGTAAAGATGAGTACTGTAGGTTTTTGTACAGCTGCTCAACAACTCCAG
TCACTGTGAGTCTCGAGCACAATAATAAACAGAATCTTCAGTCTTCAGACTGTTCATCTGCAGATACACCTGCTGTC
TGCTGTTGTCTCTGGAGATGGTAAACCGGCCTTTGACTGACTCAGAGTAGTGGATGTTGCTACTGTCATAAATAGCA
GCAACCCACTCCAGTCCTTTTCCAGGAGCCTGTCTGATCCAGTGCATCCAATAGCTACTAATTGACAGTCCAGAGGC
TGTACAGGTCAATCTGTGAGATTCTCCAGGCCTTTTAACCACTGGTTCAGATTCTGTCAGAGTCTG

>ighv10-1like.4

CCCAGTAAAGATGAGTTCTGTAGGTTTTTGTACAGCTGCTCAACCAACTCCAGTCACTGTGGCTGTCGAGCACAATA
ATAAACAGCAGAATCTTCAGTCTTCAGACTGTTCATCTGCAGATACACCTGCTGTCTGCTGTTGTCTCTGGAGATGG
TAAACCGGCCTTTGACTGACTCAGAGTAGTAGATGCTGTTACTGTCATATCTGATATACGCAATCCACTCCAGTCCT
TTTCCAGGAGCCTGTCTGATCCAGTGCATCCAGTAGCTGCTGAGTGTGAATCCAGAGGCTGTACAGGTCAATCTGTG
AGATTCTCCAGGCCTTTTAACCACTGGTTCAGATTCTGTCAGAGTCTG

>ighv10-1like.5

TCTATCAGCCCAGTAAAATTTAGCGCTGTAGGTTTTTGTGCAGCTGCTCAACCAACTGCGTTGCTGTGTGGCTGTGG
AGCACAATAATAAACAGCAGAATCTTCAGTCTTCAGACTGTTCATCTGCAGATACACCTGTTGTCTGCTGTTGTCCC
TGGAGATGGTAAACCAGCCTTTGACTGACTCAGAGTAGTACTGGGTGCTACCACTTCCACTGTAAATCCAGGCAACC
CATTCCAGTCCTTTTCCGGGAGCCTGTCTGATCCAGCTCATCCAGTTGCTGCTGAAGGTGAATCCAGAGGCTGTACA
GGTCAATCTATGAGATTCTCCAGGCCTTTTAACCACTGGTTCAGATTCTGTCAGAGTCTG

>ighv10-1like.6

GATCTTCAGCCCAGTAAAATTTTAGCGCTGTAGTTTTTGTGCAGCTGCTCAACAACTGCGTTGCTGTGTGGCTGTGG
AGCACAATAATAAACAGCAGAATCTTCAGTCTTCAGACTGTTCATCTGCAGATACACCTGTTGTCTGCTGTTGTCCC
TGGAGATGGTAACCAGCCTTTGACTGACTCAGAGTAGTACTGGGTGCTACCACTTCCACTGTAAATCCAGGCAACCC
ATTCCAGTCCTTTTCCGGGAGCCTGTCTGATCCAGCTCATCCAGTTGCTGCTGAAGGTGAATCCAGAGGCTGTACAG
GTCAATCTATGAGATTCTCCAGGCCTTTTAACCACTGGTTCAGATTCTGTCAGAGTCTG

>ighv10-1like.7

CTTTTCCAGCCCAGTAACGTTTAGTTCTGTAGGTTTTTGTACAGCTGCTCAACAACTCCAGTCACTGTGGCTCTCGA
GCACAATAATAACCAGCAGAAACTTCAGTCTTCAGACTGTTCTTCTGCAGGTACACCTGCTGTCGGCTGTTGTCTCT
GGAGATGGTAAACCGGCCTGTGACTGACTCAGAGTAGGAAGTGCCGCCACTAGCAGTATAGATCTCAGCACGGCATT
CCAGTCCTTTTCCAGGAGCCTGTCTCATCCAGTGCAAGTATTAGCTGCTGAATGTGAATCCAGAGGCTGTACAAGTC
AAACTGTGAGATTCTCCAGGCCTATTAACCACTGGTTCAGATTCTGTCAGAGTCTG

>ighv10-1like.8

TCTTTCAGTCCAGTAAAGATGAGTTCTGCAGGTTTTTGTACAGCTGCTCAACAACTCAAGTCACTGTGTCTCTCGAG
CACAATAATAAACAGCAGAATCTTCAGTCTTCAGACTGTTCATCTGCAGATACACCTGCTGTCTGCTGTTGTCTCTG
GAGATGGTAAACCGGCCTTTGACTGACTCAGAGTAGTAGATGCTACCACTGCTATCACTGATAGTAGAAACCCACTC
CAGTCCTTTTCCAGCAGCCTGTCTGATCCAAGCGTTTCCAGTAATTGCCTCCAAACCCTGAATATGTGCAGGTCAGT
CTGTGGGATTCTTCAGGCCTTTTCACTGCTGATTCAGACTCAGTCAGTGTTTG

>ighv10-1like.9

GCTTCTCATATAGAGATCAGTTGTGTTCAGTTTTTGTACAGCTCTGTAGTCTGCTGTGTCACTGTGTCTCTCGAGCA
CAATAATAAACTGCAGAGTCTTCAGTCTTCAGACTGTTCATCTGCAGATACACCTGCTGTCTGCTGTTGTCTCTGGA
ATGGTAAACCGGCCTTTGACTGACTCAGAGTAGTATTTTCCTGTCATTATCATTTTCAACCCAGGCAATCCATTCCA

GTCTTTTTCCAGGAGCCTGTCTGATCCAGTTCATCCCGTAGTCACTGATTGAATCCAGAGGCTGTACAGGTCAATCT
GTGAGATTCTCCAGGCCTTTTAACCACTGGTTCAGATTTGTCAGAGTCTG

>ighv10-1like.10

AAAATACGTCATACTTTGGCTCTTTCAGTCCAGTAAAGATGAGTATTGTAGGTTTTTGTACAGCTGCTCAACCAACT
CCAGTCACCGTGTCTCTCGAGAACAATAATAAACAGCAGAATCTTCAGTCTTCAGACTGTTCATCTGCAGATATGAC
TGCTGTCTGCTGTTGTCTCTGGAGATGGTAAACCGGCCTCTGACTGACTAGTAGTATTTGCTGCTGTCATCAGTGTA
AGTCAAGGCGATCCACTCCAGTCCTTTTCCTTTCGCAGGAAGGATGAGGAAGGAAGGCGGAAGAAAGCAACACACTA
CAATCTTCCCCACCGGTCCAGAACACTTCAAGAGCTCACCTCAGGACAGAAAGTGTGGATCACCGCAGATAAAGCTC
CTGGGACTGTTCT

>ighv10-1like.11

GAAAGTAAAGTTTTGACTTATTTTGACTTGTTGAGTTTTTGTATAGCGTTGCTCGTTCCTGTGTCACTGTGAGTCTC
AAGCACAATAATAAACAGCAGAATCTTCAGTCTTCAGACTGTTCATCTGCAGATACACCTGCTGTCTGCCATTGTCT
CTGGAGATGGTAAACCGGCCTTTGACTGACTCAGAGTAGTAGATGTAGCTACTACCATCACTGCTGATAGTAGAGAC
CCACTCCAGTCCTTTTCCAGCAGCCTGTCTGATCCAAGCGTTCCAGTAATTGCTACTGAACCCTGAATATGTGCAGG
TCAGTCTGTGGGATTCTCCAGGCCTTTTAACTGCTGGTTCAGACTCAGTCAGTGTTTG

>ighv11-2like.1

AACTGTAAGTATTTTTCAGGCTCTTTCAGCCCAGTAAAGATGAGTTCTGTAGGTTTTTCTACAGCTGCTCAACAACT
CCAGTCACTGTGGCTCTCGAGCACAATAATAAACAGCAGAATCTTCAGTCTTCAGACTGTTCATCTGCAGATACACC
TGCTGTCTGCTGTTGTCTCTGGACATGGTGAAGCGATTCTGGACTGATGTGGAATAAAGTTTAGTGCTTCCACTTGG
AGCAGAAATGTGAGAAACCCACTCCAGTCCTTTTCCTTCAGCTTGTCTGATCCAGCTGATATCAGCATCACCATCTG
ATATTCCTGCATATGTACAGGTCAGTCTGTGGGATTCTCCATGCTGCTTCACCACTGGTTCAGACTCGGTCAGAGTC
TG

>ighv11-2like.2

TACTTCAACTCAGTAAAGATGAGTTCTGTAGGTTTTTGTACAGCTGCTCAACAACTCCAGTCACTGTGACTCTCGAG
CACAATAATAACAGCAGAATCTTCAGTCTTCAGACTGTTCATCTGCAGATACACCTGCTGTCTGCTGTTGTCTCTGG
ACATGGTGAAGCGATTCTGGACTGATGTGGAATAAAGTTTAGTGCTTCCACTTGGAGCAGAAATGTGAGAAACCCAC
TCCAGTCCTTGTCCTTCAGCTTGTCTTCCAGCTGATATCAGCATCACCATCTGATATTCCTGCATATGTACAGGTCA
GTCTGTGGGACTCTCCATGCTGCTTCACCACTGGTTCAGACTCAGTCAGAGTCTG
