# Supplementary material for: Passive Immunization Delays Disease Outcome in Gilthead Sea Bream Infected With Enteromyxum leei (Myxozoa), Despite the Moderate Changes in IgM and IgT Repertoire
Source: Front Immunol. 2020 Sep 11;11:581361. doi: 10.3389/fimmu.2020.581361 (PMC7516018; doi:10.3389/fimmu.2020.581361)
Supplement: Supplementary file 4 [file Image_1.PDF]

Fig. S1

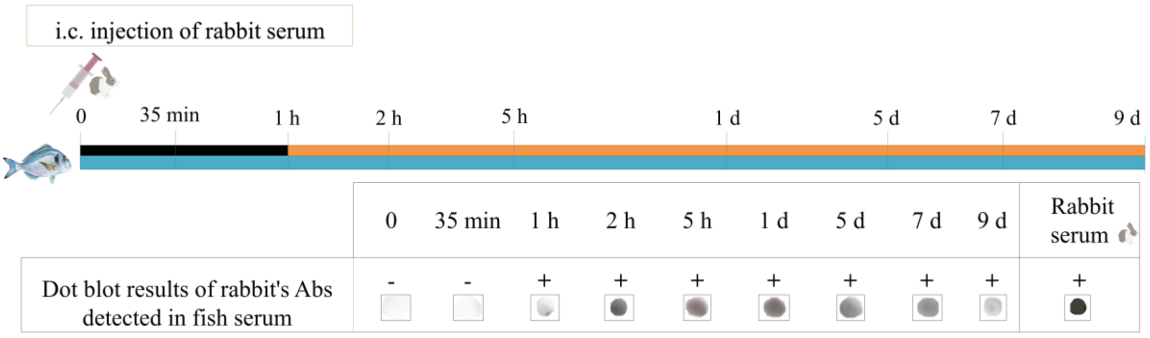

Figure S1. Rabbit IgG detection in gilthead sea bream serum after intracoelomic (i.c.) injection. Gilthead sea bream (*n* = 3) were i.c. injected with 10 µl/g of rabbit serum and 50 µl of blood were extracted after 0, 35 min, 1, 2, 5 h, 1, 5, 7 and 9 days post-injection to obtain serum. For the dot-blot, 2 µl of each serum were transferred to a nitrocellulose membrane and the membranes were blocked for 1 h with 20 mM Tris 0.5 M NaCl pH 7.4 (TBS) 5% (w/v) non-fat dry milk. Then, membranes were incubated with a 1:20,000 dilution of goat anti-rabbit-HRP (Sigma) in TBS 1% (w/v) non-fat dry milk for 1 h, washed with TBS, and visualized by chemiluminescence detection with Clarity™ Western ECL substrate (Bio-Rad) in an Amersham Imager 600 (GE Healthcare, Little Chalfont, UK). Two µl of undiluted rabbit serum were used as a positive control. The figure depicts representative images of the dot-blot results for each time point.
