# Supplementary material for: Passive Immunization Delays Disease Outcome in Gilthead Sea Bream Infected With Enteromyxum leei (Myxozoa), Despite the Moderate Changes in IgM and IgT Repertoire
Source: Front Immunol. 2020 Sep 11;11:581361. doi: 10.3389/fimmu.2020.581361 (PMC7516018; doi:10.3389/fimmu.2020.581361)
Supplement: Supplementary file 5 [file Image_2.PDF]

Fig. S2

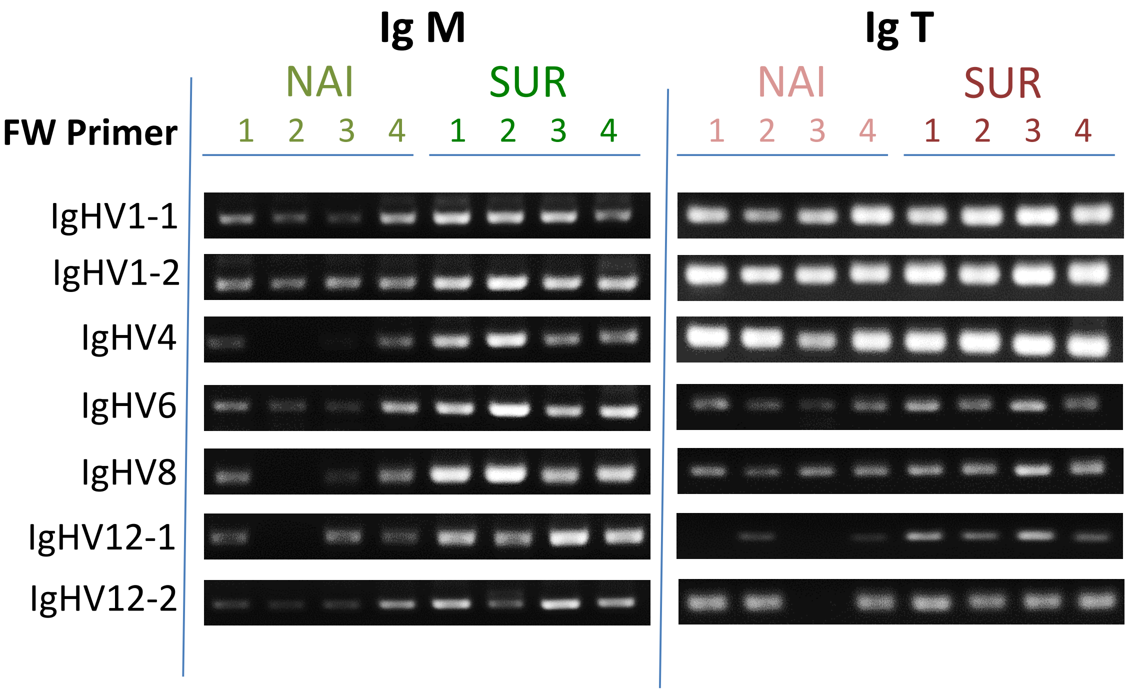

Figure S2. PCR amplification of the different combinations of forward and reverse primers in the gilthead sea bream repertoire analysis. Seven forward primers were designed to amplify the 46 VH sequences predicted from the genome. Reverse primers were specific for IgM or IgT. One PCR reaction for each of the combinations was performed on cDNA from anterior intestine of four naïve fish (NAI) and four fish that survived an *E. leei* infection and became resistant to reinfection (SUR).
